# Smoothened/AMP-Activated Protein Kinase Signaling in Oligodendroglial Cell Maturation

**DOI:** 10.3389/fncel.2021.801704

**Published:** 2022-01-10

**Authors:** Alice Del Giovane, Mariagiovanna Russo, Linda Tirou, Hélène Faure, Martial Ruat, Sonia Balestri, Carola Sposato, Francesco Basoli, Alberto Rainer, Abdelmoumen Kassoussi, Elisabeth Traiffort, Antonella Ragnini-Wilson

**Affiliations:** ^1^Department of Biology, University of Rome “Tor Vergata”, Rome, Italy; ^2^CNRS, Institut des Neurosciences Paris-Saclay, Université Paris-Saclay, Saclay, France; ^3^Department of Engineering, Università Campus Bio-Medico di Roma, Rome, Italy; ^4^Institute of Nanotechnology (NANOTEC), National Research Council, Lecce, Italy; ^5^INSERM, U1195, Université Paris-Saclay, Le Kremlin-Bicêtre, France

**Keywords:** remyelinating drugs, oligodendrocyte, differentiation, multiple sclerosis, hedgehog signaling

## Abstract

The regeneration of myelin is known to restore axonal conduction velocity after a demyelinating event. Remyelination failure in the central nervous system contributes to the severity and progression of demyelinating diseases such as multiple sclerosis. Remyelination is controlled by many signaling pathways, such as the Sonic hedgehog (Shh) pathway, as shown by the canonical activation of its key effector Smoothened (Smo), which increases the proliferation of oligodendrocyte precursor cells *via* the upregulation of the transcription factor Gli1. On the other hand, the inhibition of Gli1 was also found to promote the recruitment of a subset of adult neural stem cells and their subsequent differentiation into oligodendrocytes. Since Smo is also able to transduce Shh signals *via* various non-canonical pathways such as the blockade of Gli1, we addressed the potential of non-canonical Smo signaling to contribute to oligodendroglial cell maturation in myelinating cells using the non-canonical Smo agonist GSA-10, which downregulates Gli1. Using the Oli-neuM cell line, we show that GSA-10 promotes Gli2 upregulation, MBP and MAL/OPALIN expression *via* Smo/AMP-activated Protein Kinase (AMPK) signaling, and efficiently increases the number of axonal contact/ensheathment for each oligodendroglial cell. Moreover, GSA-10 promotes the recruitment and differentiation of oligodendroglial progenitors into the demyelinated corpus callosum *in vivo*. Altogether, our data indicate that non-canonical signaling involving Smo/AMPK modulation and Gli1 downregulation promotes oligodendroglia maturation until axon engagement. Thus, GSA-10, by activation of this signaling pathway, represents a novel potential remyelinating agent.

## Introduction

Myelin regeneration or remyelination is a fundamental repair process in the central nervous system (CNS) that is activated during pathological demyelinating events in both animal models of demyelination and humans suffering from multiple sclerosis (MS), the most common demyelinating disease of the CNS. Such spontaneous regenerative responses are observed during the early stages of the disease. However, remyelination ultimately fails in late steps, leading to loss of metabolic support normally provided by myelin to axons and, subsequently, axon degeneration and irreversible neurological disabilities. Therefore, pharmacologically induced remyelination represents great hope for CNS regenerative medicine in the context of demyelinating pathologies (Franklin and Ffrench-Constant, 2017; Plemel et al., 2017; Traiffort et al., 2020; Balestri et al., 2021; Franklin et al., 2021).

Several potential remyelinating drug candidates have been identified and are presently awaiting clinical confirmation. Further development of remyelinating therapies depends on better understanding of the mechanisms and targets that regulate myelin production. The main focus of drug discovery in CNS remyelination, thus far, has been to identify drugs that regulate myelin regenerative processes either at the level of neural progenitor cell (NPC) niches or by promoting the differentiation of oligodendrocyte precursor cells (OPCs) into oligodendrocytes (OLs) (Plemel et al., 2017; Gregath and Lu, 2018; Melchor et al., 2019; Balestri et al., 2021). Indeed, in the mouse dorsal forebrain, myelin regeneration results from OLs arising from both NPCs present in a specific germinative area called the subventricular zone (SVZ), and parenchymal OPCs (Nait-Oumesmar et al., 1999, 2008; Menn et al., 2006; Xing et al., 2014; Brousse et al., 2015). In humans, several lines of evidence also support the existence of different sources of OLs. These include the much higher turnover rate of OLs in normal-appearing white matter from MS patients than in the CNS from healthy subjects (Yeung et al., 2019), and identification of NSCs that may be recruited and fated to the oligodendroglial lineage in the human SVZ (Samanta et al., 2015). The recruitment of these various cell subsets in the site of lesion and their respective contribution to the remyelination process remain to be clarified. Moreover, in addition to the newly generated OPCs, resident OLs also appear to participate in remyelination (Franklin and Ffrench-Constant, 2017; Jäkel et al., 2019; Yeung et al., 2019).

Several promyelinating compounds have been selected in phenotypical screens based on identifying drugs upregulating MBP expression in primary OPC cultures (Deshmukh et al., 2013; Mei et al., 2014; Lariosa-Willingham et al., 2016), in epiblast stem cell-derived OPCs (Najm et al., 2015), and Oli-neuM oligodendroglial cells that stably express the gene encoding the myelin regulatory factor (MyRF) (Porcu et al., 2015). Network analysis and molecular studies have shown that the drugs identified by these screens largely overlap in their target specificity, possibly indicating a limited number of factors regulating remyelination processes (Melchor et al., 2019; Lubetzki et al., 2020; Balestri et al., 2021). Interestingly, the promyelinating drugs identified either inhibit a restricted number of cholesterol biosynthetic enzymes [e.g., emopamil-binding protein (EBP) and TM7F2 (Hubler et al., 2018; Allimuthu et al., 2019], or act *via* other targets including glucocorticoid (GR) and Smoothened (Smo) receptors (Najm et al., 2015; Porcu et al., 2015; Nocita et al., 2019). The recent finding that EBP binds to Smo and inhibits its cholesterylation independently of D8-D7 sterol isomerase activity (Qiu et al., 2021) supports the role for Smo receptor modulation in OPC maturation. However, how such Smo activity might transduce its signal to the OPC differentiation process remains unclear.

The seven-pass transmembrane Smo receptor is a well-characterized component of the Sonic Hedgehog (Shh) signaling pathway involved in both proliferation and differentiation in the embryo and early postnatal tissues (Ruat et al., 2015). In the adult brain, the delivery of exogenous Shh has been shown previously to regulate the number of oligodendroglial cells in the cerebral cortex and corpus callosum (Loulier et al., 2006), while Shh transcription has been detected in a subset of mature oligodendrocytes (Tirou et al., 2020). Smo is the key transducer of Shh signaling that is activated when Shh binds to its receptor Patched (Ptc). In the canonical pathway, Smo activation initiates a complex downstream signaling cascade resulting in the expression of glioma-associated factor 1 (Gli1) and, ultimately, transcription of Shh target genes (Petrova and Joyner, 2014; Ruat et al., 2014; Niewiadomski et al., 2019). Gli factors (Gli1-3) constitute an interconnected network of tightly correlated proteins, such as Gli2 and Gli3, which act both as activators and repressors, regulate Gli1 and each other’s expression. Moreover, Gli2 levels have been shown to correlate with decreased levels of Gli1 expression (Schmidt-Heck et al., 2015; Arensdorf et al., 2016), and promyelinating drugs, such as Clobetasol and gefitinib, upregulate Gli2 transcription (Nocita et al., 2019).

During the process of myelin regeneration, Shh/Smo signaling was found to be reactivated in demyelinating lesions. Shh signaling appeared as a positive regulator of parenchymal OPC proliferation and differentiation (Ferent et al., 2013; Sanchez and Armstrong, 2018; Laouarem et al., 2021). On the other hand, the pathway regulates NPC proliferation in the SVZ from healthy adult mice (Ferent et al., 2014; Daynac et al., 2016), whereas blockade of Gli1 is necessary to recruit a subset of NPCs that exclusively differentiate into oligodendroglial cells in mouse models of CNS demyelination (Samanta et al., 2015; Radecki et al., 2020). However, the finding that promyelinating drugs, such as Clobetasol, Halcinonide, and Flurandrenolide (Porcu et al., 2015), target GR (Najm et al., 2015) and, modulate Smo (Wang et al., 2010) and Gli2 activity (Nocita et al., 2019) in oligodendroglial cells raises the question of how Smo modulation might regulate oligodendroglial maturation.

Since Smo has also been reported to transduce Shh signaling *via* non-canonical pathways, mostly unrelated to Gli-mediated transcription (Yam and Charron, 2013; Ruat et al., 2014; Ferent and Traiffort, 2015; Schmidt-Heck et al., 2015; Sharpe et al., 2015; Akhshi and Trimble, 2021), here, we investigated the promyelinating capacity of the recently developed Smo-binding compound GSA-10 [propyl 4-(1-hexyl-4-hydroxy-2-oxo-1,2-dihydroquinoline-3-carboxamido) benzoate] (Gorojankina et al., 2013; Fleury et al., 2016). GSA-10 was previously identified in a Smo pharmacophore-based virtual screen (Gorojankina et al., 2013; Fleury et al., 2016) and belongs to a new family of Smo agonists endowed with non-canonical Shh signaling properties associated with Gli1 inhibition (Gorojankina et al., 2013; Fleury et al., 2016; Manetti et al., 2016). We compared the activity of GSA-10 in Oli-neuM differentiation with the activity of SAG, the reference Smo agonist (Wang et al., 2010; Kim et al., 2013), and Clobetasol, a GR compound that modulates Smo receptor activity (Wang et al., 2010) and promotes oligodendroglial cell differentiation (Najm et al., 2015; Porcu et al., 2015; Nocita et al., 2019). These compounds were used alone or in combination with Smo gene silencing to determine their Smo-mediated activity in the Oli-neuM cell line. By stably expressing MyRF, Oli-neuM cells differentiate into myelinating cells able to engage artificial axons in the presence of promyelinating stimuli (Porcu et al., 2015; Nocita et al., 2019). Moreover, we performed a stereotactic injection of lysophosphatidylcholine (LPC or lysolecithin) into the corpus callosum of mouse brain to determine GSA-10 efficacy in OPC recruitment toward a demyelinated lesion. Altogether, our data provide evidence that GSA-10 belongs to a novel class of Smo modulators that activate non-canonical signaling leading to Gli2 upregulation and AMPK activation, promoting MBP expression and opening new perspectives in remyelinating therapies.

## Materials and Methods

### Lysophosphatidylcholine Injection

Adult C57Bl/6 mice (Glast-CreERT2R26R-YFP, 10 weeks old) were used. The animal experiments were performed in accordance with the Council Directive 2010/63EU of the European Parliament and approved by the French ethic committees (CEEA59 and CEEA26). LPC-induced demyelination was carried out as previously described (Ferent et al., 2013) under ketamine (100 mg/kg)/xylazine (10 mg/kg)- induced anesthesia. The injection was performed at the following coordinates (to the bregma): anteroposterior (AP) +1 mm, lateral +1 mm, dorsoventral (DV) −2.2 mm. GSA-10 (100 μM) was co-injected with LPC. The mice were sacrificed 5 days post lesion (dpl). The brain was removed and frozen in liquid nitrogen, and cryostat sections (14 μm) were cut.

### Immunofluorescence in Lysophosphatidylcholine-Treated Mice

The mice, under deep anesthesia, were perfused with 4% paraformaldehyde. Brain sections were cut in the cryostat (14 μm). For immunostaining, the sections were incubated for 1 h in PBS, 0.25% Triton, and 1% BSA. Primary antibodies were incubated overnight at 4°C: rabbit anti-NG2 (1/300, AB5320; Millipore; Saint-Quentin Fallavier, France), mouse anti-GFAP (1/400, MAB360; Millipore), rabbit anti-Olig2 (1/400, AB9610; Millipore), mouse anti-S100β (1/500, AB66028; Abcam, Amsterdam, The Netherlands), mouse anti-adenomatous polyposis coli (APC) (1/600, clone CC1, OP80; Millipore), rabbit anti-Iba1 (1/500, 234003; Synaptic Systems, Uden, The Netherlands), mouse anti-Ki67 (1/200, 550609; BD Pharmigen, Le Pont de Claix, France), and mouse anti-Sox2 (1/100, MAB2018; R&D Systems, Lille, France). The sections were incubated with appropriate secondary antibody (1/200 to 1/400; Millipore, Jackson IR, Montluçon, France) for 2 h at room temperature. Staining was replicated on three or four mice. Images were acquired with a 40X objective (N.A. 0.75) using a fluorescence microscope (Leica DM2000; Leica, Wetzlar, Germany). The images were analyzed and reconstructed with ImageJ 1.39t (Freeware; NIH, New York, NY, United States).

### Cell Counting

Lysophosphatidylcholine-induced demyelination and cell count were evaluated on coronal sections obtained at the level of the lateral ventricles of 3–5 mice. Cells displaying an immunofluorescent-positive signal were counted once the extent of the demyelination had been established based on decrease in CC1 fluorescence (Bin et al., 2016) or high nuclear density. This area has been measured for each lesion and expressed in square millimeters. The quantification of antigen positive cells was expressed as percentage of the total number of DAPI-positive nuclei or as number of cells per square millimeters. The number of counted cells per lesion ranged between 500 and 6000 for DAPI-positive nuclei, 90 and 270 for Olig2^+^, 65 and 276 for Sox2^+^, 28 and 134 for NG2^+^, 9 and 153 for Ki67^+^, 126 and 278 for GFAP^+^, 47 and 158 for S100β^+^, and 131 and 359 for Iba1^+^ cells. Counting of co-localized staining was performed using ROI and multi-point ImageJ tools. Quantitative data were expressed as the mean ± standard error of the mean (SEM). Comparisons between two independent experimental groups were made by unpaired Student’s *t*-test, two-tailed. A value of *p* < 0.05 was considered statistically significant. Graphs were drawn using GraphPad Prism 5.2 (GraphPad Software, Inc.).

### Cell Culture

The Oli-neuM line (Cellosaurus ExPASy CVCL_VL76) was obtained and cultured as previously described (Porcu et al., 2015; Nocita et al., 2019), and routinely tested for contamination. The cells have been stably transfected with MyrF whose upregulation is routinely checked at each thawing in order to consistently use cells displaying MyrF at comparable levels (Porcu et al., 2015). Growth medium (GM) is based on DMEM (Corning Inc., New York, NY, United States) supplemented with 10% fetal bovine serum (FBS; Corning Inc., New York, NY, United States), 2 mM l-glutamine (Gibco*™*; Thermo Fisher Scientific, Waltham, MA, United States), 1% pen-strep (Gibco*™*), 1 mM sodium pyruvate (Gibco*™*), and 15 mM HEPES (Sigma-Aldrich, Merck KGaA, Darmstadt, Germany). The cells were maintained during growth and differentiation at 37°C in 5% CO2. A differentiation medium (DM) was GM supplemented with 1% N2 supplement (175020–01, Gibco*™*), 60 nM triiodothyronine (T3; Sigma-Aldrich), and 53.7 ng/ml progesterone (Sigma-Aldrich). The Oli-neuM cells were maintained under antibiotic selection with 500 μg/ml geneticin (G418, Gibco*™*) during both growth and differentiation.

### Compound Treatments

GSA-10 was purchased from Asinex and diluted in dimethyl sulfoxide (DMSO), stock concentration 2.5 mM, and used at a concentration of 10 μM after dose-response selection. Clobetasol (Prestw-781) was purchased from Prestwick Chemical Library^®1^. Drug stock 100 mM was diluted and used at the optimal final concentration of 10 μM (Porcu et al., 2015). SAG, Smo canonical agonist (Wang et al., 2010), was purchased from Sigma Aldrich (566660); stock and final concentrations used were, respectively, 100 and 5 μM. The final concentration was determined by dose-response analysis (data not shown). Dorsomorphin, a potent and selective AMPK inhibitor (Meley et al., 2006), was purchased from Selleckchem.com (S7306), stock concentration was 100 mM, and the concentration used was 3 μM. Dorsomorphin was solubilized in water. To obtain an equal solvent concentration, 0.5% DMSO was added in DM + dorsomorphin treatments to make drugs comparable. All the other drugs were dissolved in DMSO, used as vehicle treatment without exceeding 0.5% of the final volume. Unless otherwise stated, drug treatments were administrated in differentiation media (DM) for 48 h after 24 h from Oli-neuM seeding for fixation (IF); after 48 h from Oli-neuM seeding for protein and RNA extractions (IB and qPCR, respectively). We refer to “vehicle treatment” as the combination of DM and 0.5% DMSO max (DM + DMSO). Culturing and time of drug treatments (48 h) have been established previously to be optimal for MBP expression in Oli-neuM (Porcu et al., 2015; Nocita et al., 2019). For microfiber engage analysis, the cells were treated for 72 h according to previous established growth conditions (Nocita et al., 2019).

### Smoothened Silencing and Oli-neuM Infection

Lentiviral particles were produced by a lentivector production facility (SFR BioSciences Gerland; Lyon Sud, UMS3444/US8) from pLKO.1 plasmid-expressing shRNA targeting Smo (TRCN0000026245, MISSION^®^, Sigma), or a pLKO.1 control vector expressing nonrelevant shRNA (Gorojankina et al., 2013). For lentiviral infection, Oli-neuM cells were seeded into 12-well plates and infected after 24 h with the lentiviral particles at a multiplicity of infection of 5, in the presence of 8 μg/ml polybrene (Sigma). Puromycin (6 μg/ml) was used to select stable Oli-neuM shSmo and shControl clones, which were further allowed to differentiate, as described above. SybrGreen real-time qPCR using primers listed below in qPCR section, and IF image quantitative assay analyzing Smo expression in vehicle-treated or SAG-treated Oli-neuM shSmo vs. ShControl was used to validate knockdown efficiencies in at least 3 independent experiments.

### Crude Extract Preparation and Immunoblot Analysis

Typically, 2.75 × 10^5^ Oli-neuM cells were seeded in 6-well plates in GM media, and cells were grown to 70% confluence. Treatments, unless otherwise specified, were performed for 48 h in DM. For immunoblot analyses, the following antibodies diluted in TBS and 4% BSA were used: anti-actin (#A2066, 1:2000; Sigma-Aldrich); AbD Serotec (Bio-Rad Laboratories, Hercules, CA, United States): anti-MBP (MCA409S, 1:200); cell signaling: anti-total Ampkα (CST; #2532; 1:1000); anti-phospho Ampkα (Thr172) (CST; #2535; 1:1000). Cell extract (CE) preparation and immunoblot analyses were performed as previously described (Porcu et al., 2015; Nocita et al., 2019). Band signal intensity was estimated using the ImageJ software (version 1.8.0), and the data were plotted using GraphPad Prism 7.0 (GrahPad Software, San Diego, CA, United States) as fold change versus vehicle, arbitrarily set to 1.

### Quantitative Immunofluorescence Analysis

The cells were seeded, fixed, and processed for IF as previously described (Nocita et al., 2019). Acquisition was performed at 20 × magnification (HCX PL FLUOTAR 20 × NA 0.4) using a Leica DMI6000 B epifluorescence inverted microscope (Leica Microsystems, Wetzlar, Germany) equipped with Leica Application Suite X and Matrix Screener software (version 3.0) for automated image acquisition. Micrographs were analyzed with ScanR (version 2.1, Analysis software version 1.1.0.6; Olympus, Tokyo, Japan) for quantification and statistical analyses as previously described (Sacco et al., 2012; Porcu et al., 2015; Nocita et al., 2019). Hoechst 33,342 (Invitrogen, Thermo Fisher Scientific) staining performed used for nucleic acid quantification and nuclei detection. Rat anti-MBP (MCA409S, 1:100; Serotec), Smo [1:500; (Masdeu et al., 2006)]; phalloidin (A12380; 1:40; Thermo Fisher Scientific), and Alexa Fluor 488 or Alexa Fluor 546 conjugated secondary antibodies (Thermo Fisher Scientific) were used as indicated in the text. Image analysis was performed as previously described (Sacco et al., 2012; Porcu et al., 2015; Nocita et al., 2019). Quantitative morphological analysis was performed using either EDGE or INTENSITY module of the ScanR analysis software (Olympus) as previously described (Nocita et al., 2019). We then calculated the percentage of cell population with higher membrane area using the max Feret diameter parameter of the ScanR software. The max Feret diameter is a measure of an object size along its maximal axis. By plotting the max Feret diameter along one axis (y) and the mean intensity FITC on the other axis (X) of a scattered plot, it is possible to visualize cell distribution accordingly. Specifically, the following ScanR analysis software parameters were used for gating the cell population of interest: max Feret diameter > 250 (y)/mean intensity FITC (MBP) (x). This gate identifies the cell population with high membrane extension and MBP levels (FITC or TRITC channel according to the secondary antibody used). Cell population was also analyzed to detect populations with high MBP expression and large area according to the gate: mean intensity FITC (MBP) 130,00-40,000 (y)/area (x). Three wells per sample of three biological replicates were acquired for each experimental condition and tested for statistical significance.

### Evaluation of Cell Engagement in PS Microfibers

Cell culture chambers containing electrospun fibers were prepared as indicated in Nocita et al. (2019), UV-sterilized before use, and pre-treated with 10 μg/ml fibronectin (F0895; Sigma-Aldrich). A total of 80,000 Oli-neuM cells were seeded in a growth medium, after 24 h, the medium was changed with either DM supplemented with.5% DMSO (vehicle) or the indicated treatment(s). The cells were grown for 72 h at 37°C in 5% CO_2_. After fixation, the chambers were processed for immunofluorescence using the antibody indicated in the text. Acquisition and engagement analyses were performed as described in Nocita et al. (2019). Nuclei located in fibers characterized also by MBP expression were considered engaged. Nuclei located nearby the fibers were considered not engaged if they were at a distance not longer than 86 μm. Nuclei distant more than 86 μm from any fiber were not considered. When the distance between two fibers was shorter than 86 μm, nuclei located in between the two fibers were considered not engaged. The images were visualized and analyzed with ScanR (Olympus). 86 μm distance was estimated from the empirical observation that cells at this distance from a PS fiber never extend their membrane until to wrap the fiber. To estimate the length of MBP + processes along the fibers and the number of PS fibers associated with each Oli-neuM cell, images of the samples were taken randomly, and analyses were performed on at least 100 engaged cells, which were randomly selected per treatment. After conversion of pixels into μm [(mean pixel length)/22) * 10] (based on the objective used), the mean of the processes length was calculated using Image J tools (1.52a) version.

### Total RNA Extraction and qPCR

Following drug administration, total RNA was extracted using RNA-Solv Reagent (R6830-01; VWR) according to the manufacturer’s instructions. Typically, 2 μg of the RNA sample was retro-transcribed using the High-Capacity cDNA Reverse Transcription Kit (4368814; Thermo Fisher Scientific) according to the manufacturer’s instructions. qPCR was performed using SYBR Green Technology and the QuantStudio^®^ 3 Real-Time PCR System (Applied Biosystems^®^, Thermo Fisher Scientific). Primer pairs used with StoS Quantitative Master Mix 2X SYBR Green-ROX (GeneSpin Srl, Milan, Italy) were: Gapdh, forward (FW) 5′-CCAATGTGTCCGTCGTGGATCT-3′, reverse (RV) 5′- GTTGAAGTCGCAGGAGACAACC-3′; MBP, FW 5′-TACCCTGGCTAAAGCAGAGC-3′, RV 5′-GAGGTGGTGT TCGAGGTGTC-3′; MAL, FW 5′- CAGATCCCATCATCAGC CCC- 3′, RV 5′- TGGCTGTGTTAAGTGGGCAA-3′; OPALIN, FW 5′- CAGCTGCCTCTCACTCAACATC-3′, RV 5′- TCCCA AAGGCAGACTTCTCTCG-3′; Gli1, FW 5′-GCTGTCGGAAG TCCTATT-3′, RV 5′-ACTGGCATTGCTAAAG-3′; Gli2, FW 5′- CAACGCCTACTCTCCCAGAC-3′, RV 5′-GAGCCTTGATGTA CTGTACCAC-3′; Gli3 5′-GCAACCTCACTCTGCAACAA-3′, RV 5′- CCTTGTGCCTCCATTTTGAT-3′; Ptc, FW 5′- CCTC CTTTACGGTGGACAAA-3′, RV 5′-ATCAACTCCTCCTGCCA ATG-3′; Hip FW 5′- GAAAACGATCCCTCACCCAGCCAGAC-3′, RV 5′- GTGGGGAGAACAGCAGAGATC-3′. GAPDH was used as endogenous control. Typically, 50 ng of cDNA per sample was used per reaction. qPCR was performed in triplicate in MicroAmp Fast Optical 96-Well Reaction Plate (Applied Biosystems^®^), *n* = 3. The ΔΔCT method of relative quantification was used to determine fold change in expression. This was done by normalizing the resulting threshold cycle (CT) values of the target mRNAs to the CT values of the endogenous control Gapdh in the same samples (ΔCT = CT target–CT GAPDH), and by further normalizing to the control (ΔΔCT = ΔCT–ΔCT vehicle). Fold change in expression was then obtained (2^–ΔΔCT^) and represented in the plots using a log2 scale for ease of visualization of up/down-regulated genes.

### Statistical Methods

In studies performed in multiwell plates (immunofluorescence and qPCR), three replicates per sample were spotted in each plate, and mean values obtained from the three samples were considered as one biological replicate. The mean values ± SEM obtained from at least three biological replicates were considered for statistical analyses. Statistical analyses were performed using GraphPad Prism. Effects of each drug treatment versus its internal control (vehicle) in immunofluorescence experiments, WB, and qPCR data were analyzed by paired two-tailed Student’s *t*-test, while one-way analysis of variance (ANOVA) with Tukey’s tests (as indicated in figure legends) was performed to determine statistically significant differences among multiple single or combined treatments.

## Results

### GSA-10 Stimulates Oli-neuM Differentiation Until Axon Engagement

The observation that Gli1 downregulation is required for fating NPC cells toward myelinating oligodendrocytes (Samanta et al., 2015) suggests that the non-canonical Smo activation that is able to downregulate Gli1, might stimulate oligodendroglial differentiation. Since GSA-10 belongs to this new class of non-canonical Smo modulators, it was evaluated for its ability to promote MBP expression and Oli-neuM differentiation until axon engagement.

First, we performed a dose-response experiment (0.1, 0.3, 1, 5, 10, and 25 μM of GSA-10) to quantify MBP gene expression by quantitative RT-PCR (qPCR, Supplementary Figure 1A). In addition, immunofluorescence microscopy (IF) was used to quantify MBP levels in parallel with morphological changes in the Oli-neuM cells (Supplementary Figure 1C), since the switch of OPCs from bipolar or rhomboid morphology (undifferentiated) to enlarged and multipolar cells highly expressing MBP (differentiated) is considered to be a typical sign of OPC maturation to myelinating OLs (Snaidero et al., 2014; Nawaz et al., 2015; Zuchero et al., 2015).

The dose-response curve obtained (Supplementary Figure 1) shows that MBP levels increase at both the transcript and protein levels with increasing concentrations of GSA-10. Furthermore, the analysis of cell projections on a two-dimensional surface (gate max Ferret diameter > 250 arbitrary units) showed that high GSA-10 concentrations lead to higher number of Oli-neuM cells displaying enlarged membrane morphology compared to the vehicle condition (Supplementary Figure 1C). Altogether, these analyses show that GSA-10 treatment for 48 h promotes a dose-dependent increase in MBP, consistent with morphological changes that characterize OPC differentiation (Nawaz et al., 2015; Zuchero et al., 2015). Unless otherwise specified, a concentration of 10 μM GSA-10 was used for subsequent studies.

**FIGURE 1:**
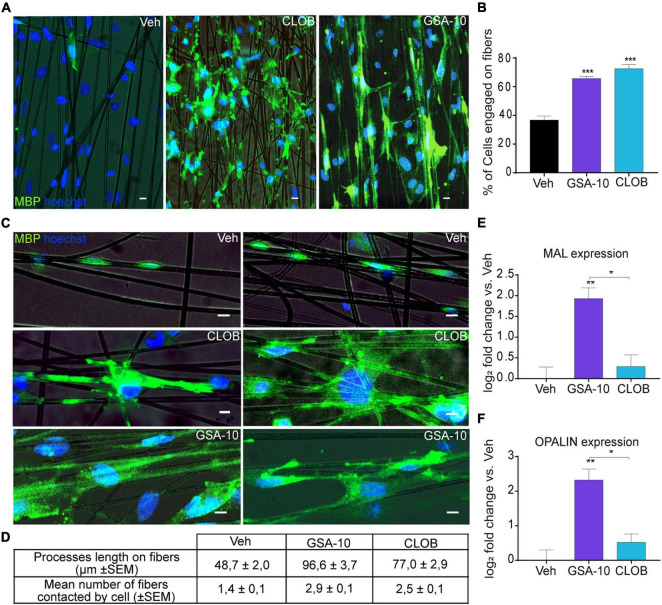
GSA-10 promotes Oli-neuM differentiation until axon engagement and compact myelin marker expression. Oli-neuM was treated with 10 μM GSA-10, 10 μM Clobetasol, or vehicle for 72 h before analysis. **(A)** Representative epifluorescence microscopy images of Oli-neuM cells, treated as indicated, in chambers containing artificial axons (aligned microfibers). Cells were stained with anti-MBP primary and Alexa 488 secondary antibodies (FITC). Hoechst (blue) = nuclei. Scale bar = 10 μm. **(B)** Quantification of fiber engagement was performed by analyzing 75 images per sample for each treatment (*n* = 3). Specifically, the percentage of engaged cells (mean ± standard error of the mean, SEM) was estimated by counting nuclei located in fibers or within a range of 86 μm from the fiber. **(C)** Enlargement of engaged cell morphology under the indicated treatments. Scale bar = 10 μm. **(D)** Quantification of membrane lengthening and number of fiber contacts by engaged cells under the indicated treatments. 100 engaged cells randomly selected per treatment were analyzed. **(E,F)** Quantitative RT-PCR analysis of MAL and OPALIN expression. Data were plotted as log2 fold change versus vehicle (which was set as 0) ± SEM (*n* ≥ 3). Two-tailed paired Student’s *t* test was performed for statistical significance versus vehicle, one-way analysis of variance (ANOVA) with Tukey’s multiple comparison test was performed to analyze statistical significance among different treatments. *p < 0.05, **p < 0.01, and ***p < 0.001.

The ability of OLs to promote axon engagement is fundamental for a promyelinating drug to be effective *in vivo* (Lee et al., 2012; Espinosa-Hoyos et al., 2018). Therefore, we cultured Oli-neuM cells in chambers containing artificial axons (electrospun aligned polystyrene, PS, microfibers of 2-5 μm diameter) in the presence of GSA-10 or the well-characterized promyelinating molecule Clobetasol. We observed that the percentage of cells engaging the artificial axons was significantly higher in the GSA-10- and Clobetasol-treated cells than in the vehicle-treated cells (Figures 1A,B). Importantly, we noted that, compared to Clobetasol, GSA-10 treatment was particularly effective in triggering lateral myelin elongation (Figures 1A,C). In addition, we quantified the length of MBP^+^ processes along fibers and the number of fibers that were associated with each Oli-neuM cell compared to Clobetasol or vehicle conditions. Both parameters were significantly increased (Figures 1C,D), indicating that GSA-10 not only induces increased axonal contact/ensheathment but also rapidly promotes cell differentiation. As mentioned above, GSA-10 was particularly efficient compared to Clobetasol. This might indicate that GSA-10 promotes a further stage of Oli-neuM maturation with respect to Clobetasol by promoting the formation of compact myelin (Snaidero et al., 2014). To corroborate this hypothesis, we analyzed the expression of two markers of compact myelin, MAL and OPALIN (Frank, 2000; Bijlard et al., 2016), in GSA- 10−, Clobetasol-, or Vehicle-treated Oli-neuM cells after a 48-h treatment. MAL is specifically upregulated during the period of active myelination and is expressed later than PLP and MBP (Schaeren-Wiemers et al., 1995; Frank et al., 1999). OPALIN is a paranodal-inner-loop glycoprotein that localizes in compact myelin (Golan et al., 2008). These late myelination markers were both upregulated in the GSA-10- but not in the Clobetasol-treated cells (Figures 1E,F).

Thus, GSA-10 not only promotes MBP expression and Oli-neuM differentiation in a dose-dependent manner but also induces Oli-neuM cells to differentiate until the stage of artificial axon engagement and the expression of myelin compaction markers.

### Smoothened Modulation by GSA-10, but Not SAG, Promotes Gli2 Upregulation and Oli-neuM Differentiation

**FIGURE 2:**
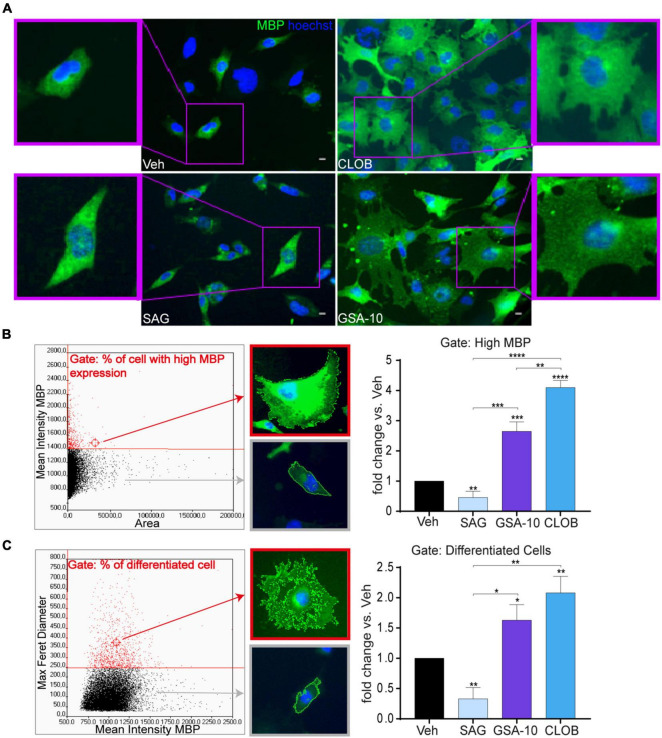
GSA-10 promotes MBP expression and Oli-neuM maturation into myelinating cells, while SAG blocks Oli-neuM differentiation. Oli-neuM was treated for 48 h with 5 μM SAG, 10 μM GSA-10, or 10 μM Clobetasol before IF analysis. **(A)** Representative images of cell morphology showing enlargement of the cell body (boxed areas). Cells were stained with anti-MBP primary and Alexa 488 secondary antibodies (FITC); Hoechst (blue) = nuclei. Scale bar = 10 μm. **(B,C)** Quantification of IF images using ScanR. **(B)** Left panel: ScanR screen detail of the gate considered: % of cells with high MBP expression [mean MBP >1400,00 (y)/area (x)]. Cell morphology in the gate and out of the gate was highlighted by a red box and a gray box, respectively. Right panel: % of the cell population within the gate compared to the vehicle condition. Values are mean ± SEM (*n* ≥ 3). **(C)** Left panel: ScanR screen detail of the gate considered: % of cells with differentiated morphology [max Feret diameter (D) > 250 (y)/mean FITC (MBP) (x)]. Fluorescent images show the cell morphology in the gate and out of the gate highlighted by a red box and gray box, respectively. Graph: % of cell population within the gate compared to the vehicle condition. Values are mean ± SEM (*n* ≥ 3). Two-tailed paired Student’s *t*-test was performed for statistical significance versus vehicle, one-way ANOVA with Tukey’s correction multiple comparison test was performed to analyze statistical significance among different treatments. *p < 0.05, **p < 0.01, ***p < 0.001, and ****p < 0.0001.

To determine how Smo modulation by GSA-10 can promote Oli-neuM differentiation, we compared the effects of GSA-10 and Clobetasol on MBP expression and cell morphology to those of the reference Smo agonist SAG (Chen et al., 2002), which binds the transmembrane-binding domain of Smo (Byrne et al., 2018) and elicits Gli1 activation (Wang et al., 2010; Ruat et al., 2014). The Oli-neuM cells were treated for 48 h with 10 μM GSA-10, 5 μM SAG, or 10 μM Clobetasol and analyzed by IF. As mentioned above, inspection of the Oli-neuM cells showed that GSA-10 and Clobetasol comparably trigger all morphological changes typical of mature OLs, while the SAG-treated cells showed a bipolar morphology, characteristic of immature oligodendroglial cells (Figure 2A). Morphological multiparametric analyses were performed by generating histograms of the scattered plot of the entire cell population based on either the mean intensity MBP (y) vs. area (x) (Figure 2B) or the max Feret diameter (y) vs. mean intensity MBP (X) (Figure 2C), as previously described (Nocita et al., 2019).

We observed that each treatment led to a different percentage of cells falling into these gates (Figures 2B,C). Indeed, GSA-10 and Clobetasol increased the percentage of cells expressing high levels of MBP by 2.5- or four-fold compared to the vehicle, respectively. In contrast, the SAG-treated cells decreased this percentage by two-fold. Similarly, we observed that GSA-10 and Clobetasol significantly increased the proportion of cells present in the max Feret diameter gate, and that SAG induced significantly less morphological differentiated cells than the vehicle.

Altogether, these data indicate that GSA-10 and Clobetasol, but not SAG, promote Oli-neuM differentiation. Specifically, GSA-10 and Clobetasol promote Oli-neuM morphological changes that are typical of mature OLs and significantly induce MBP expression, while SAG does not.

**FIGURE 3:**
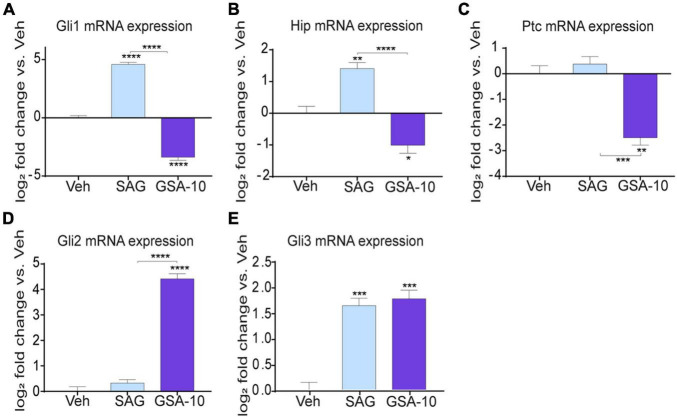
Gli, Hip, and Ptc expression analysis and Smo localization in Oli-neuM. **(A–E)** Gli1, Gli2 Gli3, Hip, and Ptc expression levels in Oli-neuM treated for 48 h with 10 μM GSA-10, 5 μM SAG, or vehicle. Data were normalized and plotted as log2 fold change. Values are mean ± SEM (*n* ≥ 3) with respect to vehicle. Two-tailed paired Student’s *t*-test was performed for statistical significance versus vehicle. One-way ANOVA with Tukey’s multiple comparison test was performed to analyze GSA-10 vs. SAG statistical significance among different treatments. *p < 0.05, **p < 0.01, ***p < 0.001, and ****p < 0.0001.

Since Smo controls the transcription of Gli1 (the main effector of canonical Hedgehog signaling), Ptc (a physiological repressor of Smo), and Hedgehog-interacting protein (Hip, a physiological repressor of Shh) (Varjosalo and Taipale, 2008; Ruat et al., 2014; Rimkus et al., 2016; Skoda et al., 2018), we further delineated how GSA-10 and SAG regulate these target genes in the Oli-neuM cells. As expected, SAG was found to significantly upregulate Gli1 and Hip expression, but it was not able to regulate Ptc compared to the vehicle (Figures 3A–C). In contrast, GSA-10 significantly inhibited all the target genes (Figures 3A–C). Moreover, since Gli transcription factors are tightly correlated in an interconnected network and because Gli2 and Gli3 can act both as activators and repressors affecting Gli1 levels in other cell types (Schmidt-Heck et al., 2015), we also analyzed Gli2 and Gli3 expression under GSA-10 and SAG treatments. Gli2 and Gli3 expression was significantly increased by GSA-10 (Figures 3D,E). In contrast, SAG had no activity in Gli2, but it stimulated Gli3 expression like GSA-10. These data, thus, suggest that Gli2 upregulation may correlate with the promyelinating activity of GSA-10.

### Smoothened Activity Is Necessary for MBP Expression and Oli-neuM Differentiation After Myelin Regulatory Factor Expression

To conclusively establish the requirement of Smo modulation for Oli-neuM differentiation, we performed Smo gene silencing in the Oli-neuM cells and evaluated MBP protein levels, cell differentiation, and late myelin gene expression (MAL and OPALIN) after SAG and GSA-10 treatment. To this end, Oli-neuM cells were stably transfected using pLKO.1 lentiviral Smo shRNA (shSmo) or a nonrelevant shRNA-expressing pLKO.1 vector (shControl; Gorojankina et al., 2013).

**FIGURE 4:**
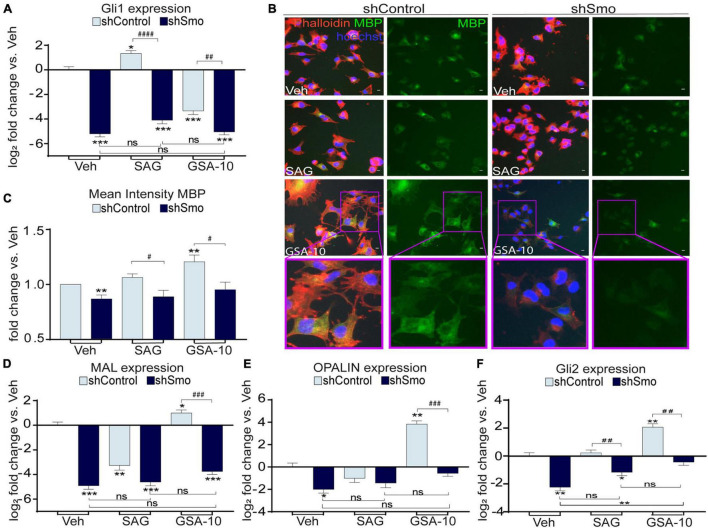
Smo silencing decreases GSA-10-induced MBP, MAL, OPALIN, Gli2 gene expression, and abrogates GSA-10 activity in Oli-neuM differentiation. **(A)** Gli1 gene expression levels. Values were normalized and plotted as log2 fold change and are mean ± SEM (*n* ≥ 3) with respect to vehicle shControl. **(B)** Representative IF images of shSmo or shControl cells under the indicated treatments and stained with anti-MBP primary and Alexa 488 secondary antibodies (green); Phalloidin (red); Hoechst (blue) = nuclei. Scale bar = 10 μm. Cell magnifications are shown in boxed area. **(C)** Quantitative analyses of IF cell images. Mean intensity of MBP ± SEM (*n* ≥ 3) was plotted as fold change vs. shControl vehicle. **(D–F)** MAL, OPALIN, and Gli2 gene expression levels. Values were normalized and plotted as mean log2 fold change vs. vehicle shControl ± SEM (*n* > 3). Two-tailed paired Student’s *t*-test was performed for statistical significance vs. shControl vehicle (* labeled) and to compare shControl vs. shSmo (# labeled) in each treatment. One-way ANOVA with Tukey’s multiple comparison test was performed to analyze statistical significance between the different treatments. *p < 0.05, **p < 0.01, ***p < 0.001; #*p* < 0.05, ##*p* < 0.01, ###*p* < 0.001, and ####*p* < 0.0001; ns, non significant.

SAG has been previously shown to induce Smo internalization that leads to apparent increase in Smo-associated fluorescent staining in the cytoplasm of cultured cells (Chen et al., 2004; Wang et al., 2010). In agreement with this observation, SAG, unlike GSA-10, increased the immunofluorescent Smo signal in the cytoplasm of Oli-neuM cells. However, our quantifications also revealed an increased amount of Smo protein and transcripts (Supplementary Figures 2A,B). Therefore, we used the SAG condition in order to validate the efficiency of shSmo. As expected, after shSmo lentivirus transfection, we observed a significant reduction in Smo protein levels (Supplementary Figures 3A,B) and gene expression (Supplementary Figure 3C) compared to shControl in both vehicle- and SAG-treated Oli-neuM cells. Thus, these data show that Smo is effectively silenced in this cell line, and that shSmo displays an apparent lower ability to regulate Smo protein than Smo RNA, which might be related to a slow turnover of the protein. To further confirm Smo gene silencing, we analyzed Gli1 gene expression in the shSmo and shControl Oli-neuM cells upon SAG or GSA-10 treatment (Figure 4A). As expected, Oli-neuM shControl showed significant upregulation of Gli1 after SAG treatment, while the GSA-10-treated shControl cells displayed significant downregulation of Gli1. SAG-induced increase in Gli1 was blocked by shSmo (Figure 4A), indicating effective Smo silencing. Moreover, GSA-10 lost its ability to downregulate Gli1 at a level significantly different from that of the vehicle in the presence of shSmo, showing that GSA-10-mediated inhibition of Gli1 also depends on Smo (Figure 4A).

We compared MBP levels in the shSmo vs. shControl Oli-neuM cells under GSA-10, SAG, or vehicle treatment. Quantitative analysis of MBP IF staining (Figures 4B,C) showed that under all the treatment conditions, shSmo induced a significant decrease in both MBP^+^ signal and Oli-neuM differentiation reflected by the modification of cell morphology (Figure 4B). In addition, MAL and OPALIN gene expression was significantly downregulated in the GSA-10-treated shSmo Oli-neuM cells compared to the shControl cells (Figures 4D,E). SAG did not stimulate MAL and OPALIN expression either in shSmo or in shControl. Finally, Smo silencing, per se, significantly inhibited the expression of these myelin genes. Moreover, since we showed the capacity of GSA-10 to upregulate Gli2, we finally evaluated how Smo gene silencing influences GSA-10 effect on Gli2 gene expression. GSA-10-induced Gli2 upregulation was completely abrogated in shSmo compared to shControl cells (Figure 4F).

We conclude that Smo is required for Oli-neuM differentiation into myelinating cells, and that only GSA-10-mediated activation of Smo is able to upregulate myelin proteins such as MBP, MAL, and OPALIN. Furthermore, GSA-10-induced Gli1 and Gli2 gene transcription also requires Smo.

### GSA-10-Mediated Smoothened/AMP-Activated Protein Kinase Activation Induces MBP, MAL, and OPALIN Expression

**FIGURE 5:**
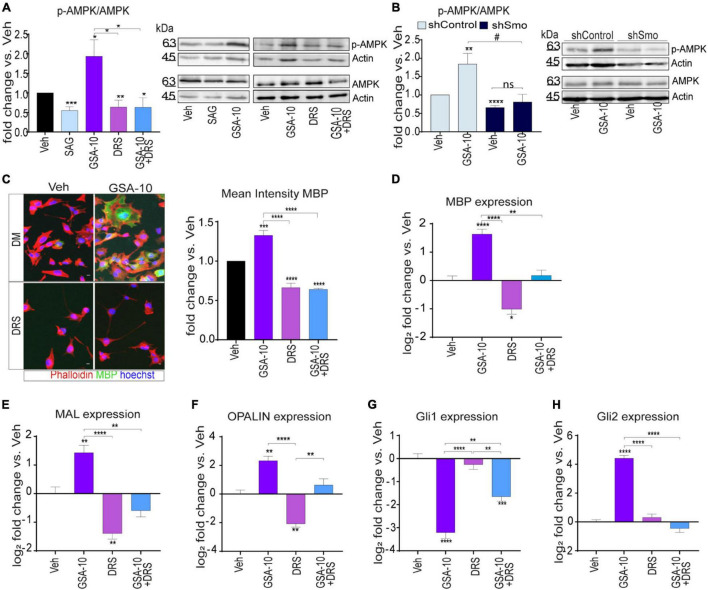
AMPK signaling is required for Oli-neuM differentiation and Gli2 gene expression. **(A)** Immunoblot analysis of Oli-neuM cell extracts after treatment with 5 μM SAG, 10 μM GSA-10, 3 μM Dorsomorphin (DRS), or alone or in combination with GSA-10. Left panel: Quantitative analysis of protein levels: anti-AMPK (AMPK) or phospho-AMPK (pAMPK) antibody. Anti-actin antibody was used to normalize sample loading (*n* ≥ 3). Signal intensity was estimated using ImageJ software tools, and values ± SEM were plotted as the fold change of p-AMPK/AMPK. Right panel: representative image of immunoblots used for quantitative analysis. **(B)** Immunoblot analysis of shSmo vs. shControl extracts after treatment with 10 μM GSA-10 or vehicle. Left panel: Quantitative analysis of protein levels: anti-AMPK (AMPK) or phospho-AMPK (pAMPK) antibody. Quantification was performed as in panel **(A)**. Values ± SEM (*n* ≥ 3) were plotted as the fold change of p-AMPK/AMPK. Right panel: representative image of immunoblots used for quantitative analysis. **(C)** Quantitative IF images analysis of MBP in Oli-neuM. Left panel: Typical treated cell images, stained with anti-MBP primary and Alexa 488 secondary antibodies (green); Phalloidin (red); Hoechst (blue) = nuclei. Scale bar = 10 μm. Right panel: Mean FITC (MBP) intensity was plotted as mean ± SEM (*n* ≥ 3). Data were normalized with respect to vehicle, arbitrarily set to 1, and plotted as fold induction versus vehicle. **(D–H)** Quantitative RT-PCR analysis of MBP, MAL, OPALIN, Gli1, and Gli2 expression under GSA-10, DRS, or their combination. Values ± SEM (*n* ≥ 3) were plotted as log2 fold change vs. vehicle (which is equal to 0). Statistical significance: two-tailed Student’s *t*-test was performed for treatment vs. vehicle (vs. shControl in panel **(B)**), ANOVA one-way with Tukey’s multiple comparison test was performed to determine significance among different treatments. *p < 0.05, **p < 0.01, ***p < 0.001, and ****p < 0.0001; #*p* < 0.05; ns, non significant.

As mentioned above, Smo can modulate Gli1-independent intracellular signals in different cellular systems (Ruat et al., 2014; Rimkus et al., 2016). Here, we focused on the analysis of the AMPK signaling pathway, since this pathway was recently found to restore the capacity of aged OPCs to differentiate (Neumann et al., 2019). Immunoblot analyses of cell extracts from the Oli-neuM cells treated for 48 h with 10 μM GSA-10 or 5 μM SAG showed a significant increase in AMPK phosphorylation under GSA-10 treatment, while SAG significantly decreased the pAMPK/AMPK ratio (Figure 5A).

To determine if AMPK phosphorylation depends on Smo activation under our experimental conditions, we evaluated the ability of GSA-10 to induce AMPK phosphorylation in the shSmo cells compared to shControl cells (Figure 5B). GSA-10 was unable to promote AMPK phosphorylation in the shSmo cells, while it was still efficient in the shControl cells.

To further investigate AMPK activation and its relevance in MBP induction upon GSA-10 treatment, we used the selective phospho-AMPK inhibitor Dorsomorphin (DRS; Figure 5A) (Lo et al., 2019; Sun et al., 2021) in IF (Figure 5C) and quantitative RT-PCR analyses (Figure 5D) after 48 h treatment. As expected, DRS alone induced decrease in AMPK phosphorylation (Figure 5A). Moreover, in the presence of DRS, GSA-10 failed to increase AMPK phosphorylation (Figure 5A). Under both conditions, a highly significant reduction in MBP protein (Figure 5C) and MBP mRNA (Figure 5D) levels was observed. In agreement with these observations, the cells were unable to adopt the morphology characteristic of differentiated cells (Figure 5C). Along the same line, DRS prevented GSA-10-mediated MAL and OPALIN upregulation (Figures 5E,F), decreased by half GSA-10-induced Gli1 downregulation, and fully abrogated GSA-10-induced Gli2 upregulation (Figures 5G,H).

These data indicate that by modulating the Smo/AMPK pathway, GSA-10 promotes Oli-neuM morphological changes typical of mature oligodendroglial cells by stimulating Gli2 gene expression as well as MBP and compact myelin gene expression.

### GSA-10 Increases the Recruitment of New Differentiated Oligodendrocytes in Demyelinated Areas *in vivo* After Lysophosphatidylcholine Injection Into the Corpus Callosum

**FIGURE 6:**
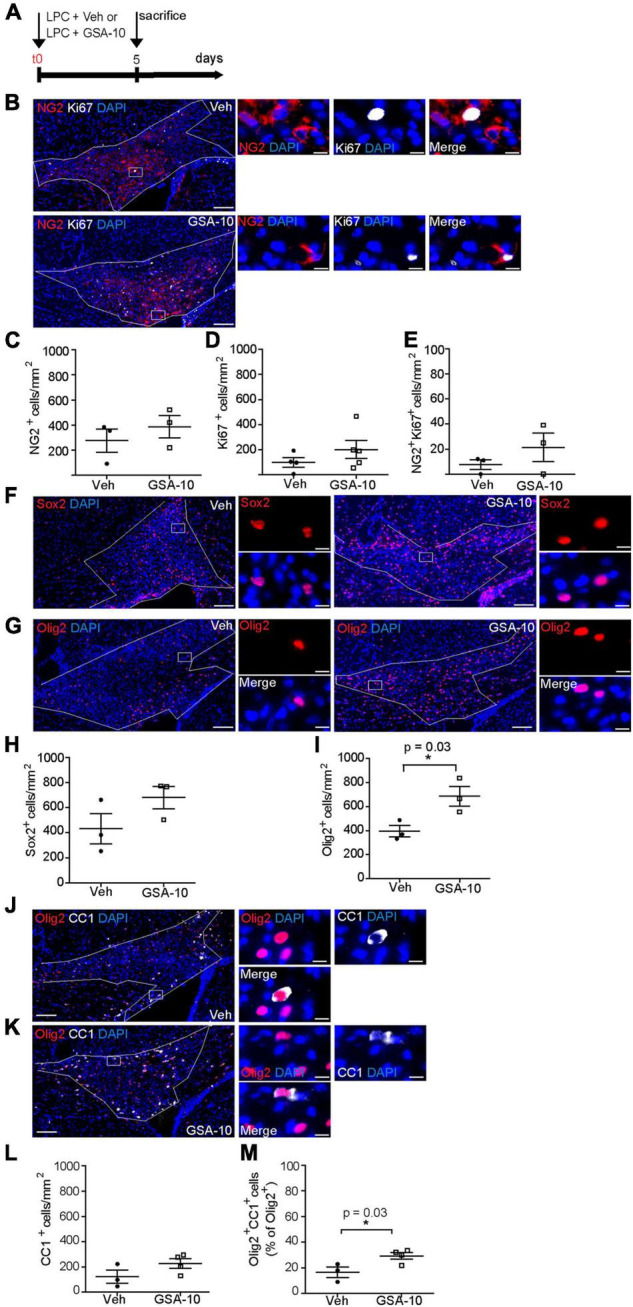
GSA-10 increases Olig2^+^ cells during remyelination of the corpus callosum. **(A)** Scheme of LPC-induced focal demyelination and GSA-10 treatment. LPC was injected into the corpus callosum of adult mice in the presence of vehicle (Veh) or the quinolinone GSA-10. Mice were perfused 5 days post-lesion to allow OPC recruitment. **(B)** Distribution of Ki67 (white) proliferation marker together with the proteoglycan-NG2 marker (red) inside the lesioned corpus callosum of Veh and GSA-10 treated brains. **(C–E)** GSA-10 does not modify the density of **(C)** NG2^+^, **(D)** Ki67^+^, and **(E)** NG2^+^Ki67^+^ cells inside the lesion. Mean ± SEM, *n* = 3–4 experimental replicates. **(F,G)** Distribution of the oligodendroglial markers **(F)** Sox2 and **(G)** Olig2 (red) inside the lesioned corpus callosum of Veh or GSA-10 treated brains. **(H,I)** GSA-10 increases the density of Olig2^+^ cells **(I)** (*p* = 0.01) and does not significantly modify the density of Sox2^+^ **(H)** cells inside the lesion. **(J,K)** Distribution of CC1 (white) marker labeling-differentiated oligodendrocytes together with the Olig2 marker (red), inside the lesioned corpus callosum of Veh and GSA-10-treated brains. **(L,M)** GSA-10 increases the density of CC1^+^Olig2^+^ cells and their percentage in the Olig2 population inside the lesion (*p* = 0.03). Mean ± SEM, *n* = 3–5 experimental replicates. White squares are magnified on the right **(B,F,G,J,K)** of the main panels, showing positive cells in merge and single channel with the nuclear marker DAPI (blue). Scale bar: **(B,F,G)** = 100 μm; all magnifications = 10 μm. *p < 0.05.

We reasoned that the ability of GSA-10 to activate a non-canonical Smo signaling pathway resulting in Gli1 downregulation and Gli2 upregulation might promote the recruitment of NPCs, as recently shown for the Gli1 antagonist GANT-61 (Samanta et al., 2015; Namchaiw et al., 2019; Radecki et al., 2020). To validate this idea, a stereotactic injection of LPC was performed into the corpus callosum in the presence of GSA-10 in order to determine the effects of non-canonical Smo modulation on the recruitment of oligodendroglial cells upon CNS demyelination. GSA-10 or the corresponding vehicle was co-injected with LPC, and the brains were analyzed 5 days post lesion (dpl) to allow for oligodendroglial cell recruitment into the demyelinated area (Figure 6A). First, we investigated the effect of GSA-10 on OPC proliferation using an antibody directed against the NG2 proteoglycan and the antibody Ki67 as a marker of cell proliferation (Figure 6B). Cell quantification indicated that GSA-10 does not induce any OPC proliferation as shown by the unmodified density of NG2^+^ cells (386.7 ± 88.7 vs. 277 ± 92.8 cells/mm^2^ or 5.2 ± 0.6 vs. 3.9 ± 0.3% of DAPI^+^ cells; Figure 6C), NG2^+^ proliferation capacity (7.5 ± 4.9 vs. 4.5 ± 3.2% of Ki67^+^ cells; Figure 6E), and whole density of proliferating cells inside the lesion (341.5 ± 77.3 vs. 277 ± 92.8 cells/mm^2^ or 5.8 ± 2.3 vs. 4.2 ± 1.6% of DAPI; Figure 6D) when compared to the vehicle (Figures 6C–E). Along the same line, although the density of cells expressing the transcription factor Sox2, previously reported to play an important role in OPC recruitment by maintaining them in a proliferative state (Zhao et al., 2015), appeared to increase under GSA-10 treatment, but the difference was not significant (680.7 ± 88.8 vs. 432.3 ± 120.7 cells/mm^2^ or 18.9 ± 1.3 vs. 12.2 ± 2.8% of DAPI^+^ cells, Figures 6F,H). We further used Olig2, a well-known marker of the whole oligodendroglial lineage. We observed a significant increase in the density of Olig2^+^ cells inside the lesion in GSA-10- compared to vehicle-treated mice (687 ± 82 vs. 396 ± 47 cells/mm^2^, *p* = 0.036 or 15.1 ± 1.3 vs. 9.4 ± 0.8 % of DAPI^+^ cells, *p* = 0.01; Figures 6G,I), indicating that GSA-10 regulates oligodendroglial recruitment toward the lesion independently of cell proliferation. The increase in Olig2^+^ cells prompted us to investigate a putative increase in the number of differentiated oligodendrocytes in this cell population. Therefore, we analyzed CC1 expression as a marker of the cells in the same animals (Figures 6J,K). Cell quantification showed increase in both the density of CC1^+^ cells and the percentage of Olig2^+^ cells co-expressing CC1 (122.3 ± 52.5 vs. 226.3 ± 37.7 cells/mm^2^ or 16.5 ± 4.1 vs. 29.3 ± 2.6% of Olig2^+^ cells, *p* = 0.03), indicating that GSA-10 promotes the differentiation of oligodendroglial progenitors (Figures 6L,M).

**FIGURE 7:**
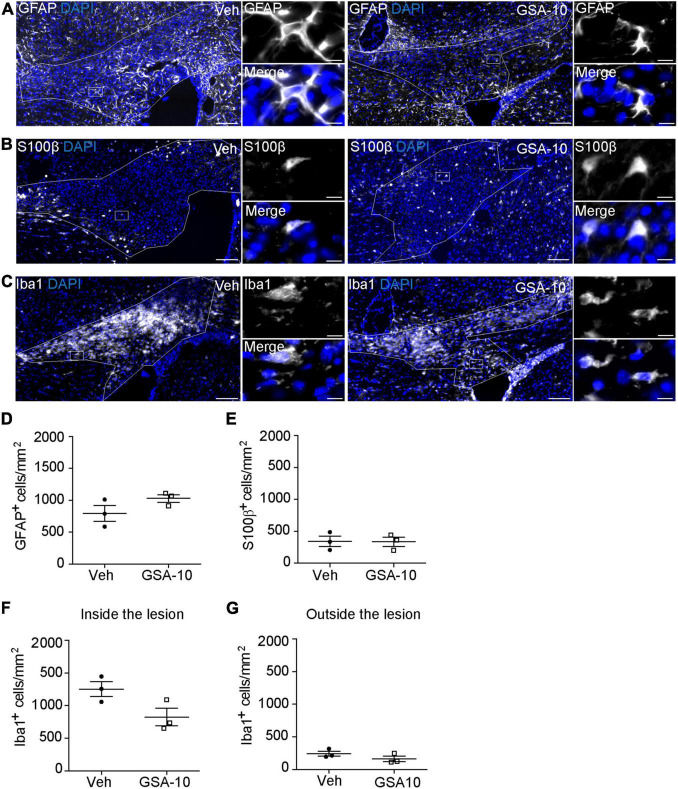
GSA-10 does not affect astrogliosis and microgliosis in the lesioned corpus callosum. **(A–G)** Distribution of **(A)** GFAP and **(B)** S100β astroglial markers or **(C)** Iba1 microglial marker inside the lesioned corpus callosum of vehicle (Veh) and GSA-10 treated mouse brains. GSA-10 does not modify significantly the density of **(D)** GFAP^+^, **(E)** S100β ^+^, or **(F)** Iba1^+^ cells inside the lesion. Most S100β^+^ cells display a cytoplasmic labeling characteristic of astroglial cells. Moreover, as observed inside the lesion, the number of Iba1^+^ microglia cells was not modified by GSA-10 outside the lesion **(G)**. Mean ± SEM, *n* = 3–4 experimental replicates. White squares are magnified on the right side of the main panel, showing positive cells in merge and single channel with the nuclear marker DAPI (blue). Scale bar: **(A–C)** = 100 μm; all magnifications = 10 μm.

We then asked whether GSA-10 could affect astrogliosis and microgliosis in the lesion. To this end, we analyzed the expression of the astroglial markers GFAP (Figure 7A) and cytoplasmic S100β (Figure 7B), and the microglial marker Iba1 (Figure 7C). GSA-10 did not change the density of either GFAP^+^ (1029 ± 59.2 vs. 794.3 ± 1 GFAP^+^ cells/mm^2^, or 19.2 ± 1.9 vs. 18.8 ± 1.9% of DAPI^+^ cells; Figure 7D) or S100β^+^ (335 ± 70.1 vs. 343 ± 80.9 S100β^+^cells/mm^2^, or 9.05 ± 0.7 vs. 10.1 ± 2.5% of DAPI^+^ cells; Figure 7E) astrocytes, or the number of Iba1^+^ microglia (826.3 ± 134.7 vs. 1254 ± 112.6 cells/mm^2^, or 25.6 ± 2.8 vs. 30.5 ± 2.3% of DAPI^+^ cells; Figures 7F,G) when compared to the vehicle condition.

Altogether, the data show that GSA-10 promotes oligodendroglial cell recruitment without inducing progenitor proliferation and does not affect the number of astrocytes and microglial cells inside the lesion.

## Discussion

Although previously reported arguments have supported the hypothesis that Smo-mediated Shh signaling may control oligodendroglial differentiation, the molecular mechanisms involved are still unclear (Ruat et al., 2014; Del Giovane and Ragnini-Wilson, 2018). Using *in vitro* and *in vivo* complementary approaches, we show here that a non-canonical modulator of the Smo receptor, GSA-10, is a potent activator of the maturation of the oligodendroglial cell line Oli-neuM into myelinating cells, and that it promotes the recruitment and differentiation of Olig2^+^oligodendroglial cells into the demyelinated CNS areas. Compared to other pharmacological modulators of Smo activity, GSA-10 displays the property of promoting Oli-neuM differentiation up to the stage of engaging artificial axons and the expression of two markers of compact myelin, MAL and OPALIN, by increasing contact with- or ensheathment of the fibers. Importantly, gene silencing experiments clearly indicate that Smo modulation is required during the differentiation of Oli-neuM cells induced by GSA-10. Moreover, we provide evidence for the involvement of Smo-mediated AMPK signaling activation in the capacity of GSA-10 to stimulate morphological changes and increase in MBP, MAL, and OPALIN expression associated with Oli-neuM differentiation. Smo/AMPK signaling involves the ability of GSA-10 to decrease Gli1 and increase Gli2 transcription. Altogether, our data provide arguments supporting a higher level of complexity than initially thought in how Shh signaling may respond to demyelination.

Besides GSA-10, other small molecules are able to activate Smo/Gli signaling and oligodendroglial maturation, such as compounds belonging to the glucocorticoid class, namely Clobetasol, Halcinonide, and Flurandrenolide (Wang et al., 2010; Porcu et al., 2015; Del Giovane and Ragnini-Wilson, 2018). In contrast to these classes of Smo modulators, and to the reference agonist SAG (Wang et al., 2010; Kim et al., 2013), osteogenic quinolone derivatives, represented by the small molecule GSA-10, have not yet been evaluated in the context of myelin regeneration. Quinolone derivatives have been identified previously *via* the generation of a pharmacophoric model of Smo agonists (Gorojankina et al., 2013; Manetti et al., 2016). The use of the selective Smo agonist GSA-10 in the context of this study represents a critical tool to dissect the role of Smo receptor signaling in the last step of oligodendroglial maturation from MyRF expression until axon engagement. The approach also revealed the requirement for Smo-mediated activation of AMPK signaling in oligodendroglial maturation.

At the cellular level, we show that GSA-10 treatment promotes not only a dose-dependent increase in MBP, allowing Oli-neuM cells to mature until the ensheathment of polystyrene microfibers, currently used in drug validation studies as *in vitro* axon surrogates (Lee et al., 2012; Allimuthu et al., 2019; Nocita et al., 2019; Starost et al., 2020; Balestri et al., 2021), but also lateral membrane lengthening and compact myelin formation. Importantly, Smo activation by GSA-10, but not by SAG, leads to AMPK phosphorylation and Gli2 upregulation, which together correlate with the increase in MBP expression *via* a pathway that requires Smo, AMPK activation, and Gli1 downregulation. Smo gene silencing demonstrates that AMPK-mediated signaling and Gli2 upregulation are generated by GSA-10 modulation of Smo activity, and that SAG fails to modulate these responses. In addition, our experiments using DRS lead us to propose that GSA-10-mediated Gli2 upregulation requires AMPK activation. Since Gli1 ablation has been proposed previously to increase Gli2 expression *in vivo*, as especially shown in NSCs upon CNS demyelination (Radecki et al., 2020), we may hypothesize that the partial impairment of GSA-10-mediated Gli1 downregulation induced by DRS in Oli-neuM cells may subsequently prevent Gli2 upregulation. This hypothesis would further corroborate the importance of relative expression levels of the two transcription factors in the context of myelin production (Radecki et al., 2020). However, this remains to be verified. Altogether, these data further support the observation that different Smo agonists, presumably by activating Smo at distinct sites, can activate separate Smo-dependent pathways (Gorojankina et al., 2013; Fleury et al., 2016; Akhshi and Trimble, 2021). Clearly, AMPK phosphorylation, following GSA-10-induced Smo activation, is important for both basal MBP expression and morphological changes occurring during Oli-neuM differentiation into myelinating cells, since dorsomorphin-treated Oli-neuM cells display lower MBP expression than cells treated with the vehicle and no differentiated morphology.

Another major finding of our study comes from the comparison of the remyelinating properties of GSA-10 and Clobetasol, the first glucocorticoid and Smo receptor modulator proposed to enhance remyelination in patients (Najm et al., 2015; Porcu et al., 2015). We observed that GSA-10 not only induces MBP expression as well as Oli-neuM engagement of artificial axons, similarly to Clobetasol (Porcu et al., 2015; Nocita et al., 2019), but also stimulates a further stage of oligodendroglial cell maturation, namely, membrane lateral lengthening. Further confirming the differences between Clobetasol and GSA-10 activity in late maturation stages, only GSA-10 promotes expression of the late markers of myelin compaction MAL and OPALIN. AMPK stimulation by GSA-10 might be the leading event that drives the stimulation of these genes and, thereby, compact myelination.

How GSA-10 promotes selective Smo-mediated AMPK phosphorylation signaling remains to be determined. As previously mentioned, GSA-10 has been identified through the virtual screening of a library of compounds in a pharmacophoric model of Smo agonists, such as SAG. SAG and GSA-10 were proposed to stabilize different active forms of the Smo receptor (Gorojankina et al., 2013). The latter can indeed adopt several conformations in the cell cytoplasm and primary cilium (Rohatgi et al., 2009; Rominger et al., 2009; Wilson et al., 2009; Yang et al., 2009; Belgacem and Borodinsky, 2011; Roudaut et al., 2011; Gorojankina et al., 2013; Ruat et al., 2014). SAG is proposed to stabilize a form addressed to the primary cilium and leads to activation of the Shh pathway *via* transcription of Shh-related genes. In contrast, GSA-10 is suggested to stabilize a form that does not translocate to the cilium (Gorojankina et al., 2013). One possibility is that GSA-10 might stabilize the Smo receptor in early endosomes and, thereby, increase the ability of Smo to interact with the liver kinase b1 (Lkb1), which is known to promote AMPK activation (Teperino et al., 2012, 2014). In agreement with this hypothesis, the Lkb1/AMPK axis was found to act downstream of Smo activation and inhibit adipocyte differentiation (Fleury et al., 2016). In support of the hypothesis that Smo-mediated AMPK pathway modulation is important for CNS remyelination is the finding that metformin, which is able to activate AMPK signaling, has been previously reported to restore the differentiation capacity of aged OPCs (Neumann et al., 2019).

The role of Smo modulation during remyelination processes was previously studied *in vivo*. Specifically, it was shown that the adenovirus-mediated transfer of Shh or its physiological antagonist Hip close to LPC-induced lesions of the corpus callosum accelerated or prevented remyelination, respectively (Ferent et al., 2013). Along the same line, the Smo reference agonist SAG has been reported recently to both directly promote the proliferation of parenchymal OPCs and indirectly induce the differentiation of these progenitors by regulating microglia response to demyelination in the LPC model (Laouarem et al., 2021). On the other hand, the blockade of the main effector of the canonical Shh signaling pathway, Gli1, has been shown to promote the recruitment of a subset of NPCs located in the SVZ bordering the lateral ventricles and exclusively fated to the oligodendroglial lineage (Samanta et al., 2015).

The increase in Olig2-expressing cells induced by GSA-10 when it is concomitantly injected with LPC into the corpus callosum shows the ability of this drug to promote oligodendroglial cell recruitment into demyelinated lesions *in vivo*. Most importantly, GSA-10 appears to accelerate and promote the differentiation of oligodendroglial progenitors into CC1^+^-differentiated oligodendrocytes, indicating that GSA-10 acts in several steps of the remyelination process, i.e., recruitment of progenitors and their differentiation into oligodendrocytes. In addition, our *in vitro* experiments provide arguments supporting that GSA-10 induces myelin gene expression as well as axon engagement. Although genetic tracing of the recruited progenitors and progeny would be required to accurately delineate the source of the newly generated oligodendrocytes, the present data nevertheless provide arguments supporting the hypothesis that both NPCs and OPCs might be recruited *via* the non-canonical activation of Smo by GSA-10. Indeed, GSA-10 fails to significantly promote parenchymal OPC proliferation as attested by the unmodified number of NG2^+^ cells detected in the lesion, which may be the result of the accelerated differentiation of OPCs and/or the recruitment of SVZ-derived NSC progeny. The latter hypothesis recalls the activity attributed to the antagonist of the Gli transcription factors, GANT-61. Indeed, GANT-61 was reported to exclusively recruit a subset of SVZ-derived Gli1-expressing NPCs fated to the OL lineage and to be devoid of effects on parenchymal OPCs (Samanta et al., 2015). Like the pharmacological blockade of Gli1, its genetic inhibition was found to be crucial to improve the functional outcome of animals in a relapsing/remitting EAE model and to promote remyelination in the cuprizone model of CNS demyelination (Samanta et al., 2015). Due to the well-characterized capacity of GSA-10 to downregulate Gli1 (Gorojankina et al., 2013; Fleury et al., 2016; Akhshi and Trimble, 2021; and our present data), we hypothesize that GSA-10 may act in the same way as GANT-61 on the recruitment of SVZ-derived NPCs. Consequently, our observation that Sox2 tends to increase, albeit not significantly, upon GSA-10 local administration into the lesion probably may reflect the contribution of Sox2 to the expansion of OPCs and/or its ability to prime oligodendroglial progenitors to eventually undergo differentiation, as previously reported (Zhao et al., 2015).

An additional and original aspect of our study is that GSA-10 stimulates Gli2 and Gli3 expression, and that it downregulates Gli1. GSA-10-induction of Gli2 and Gli3 expression was not observed upon treatment of murine mesenchymal C3H10T1/2 cells (Gorojankina et al., 2013). This suggests a different regulation of Smo-mediated signals in oligodendroglial cells compared to mesenchymal cells.

Two other promyelinating drugs, namely, Clobetasol and Gefitinib, also stimulate Gli2 in Oli-neuM. In this case, we have shown that Gli2 expression depends on RxRγ expression, since RxRγ gene silencing abrogates Gli2 expression in Oli-neuM under Clobetasol or Gefitinib treatments (Nocita et al., 2019). The recent demonstration that Sox17-induced oligodendrocyte regeneration in adult myelin lesions occurs by suppressing lesion-induced Wnt/beta-catenin signaling through an increase in Shh/Smo/Gli2 activity (Ming et al., 2020) supports the importance of Gli2 upregulation for the differentiation program under Gli1 downregulation. Along the same line, Gli2 has been reported to promote the differentiation of Gli1-null NPCs into OLs in a cuprizone model of demyelination. In addition, the genetic ablation of Gli1 in NPCs was reported to increase Gli2 expression, while the loss of both factors was found to decrease NPC recruitment and the differentiation of their progeny (Radecki et al., 2020). Therefore, we suggest that by downregulating Gli1 and subsequently upregulating Gli2, the non-canonical Smo agonist GSA-10 might open promising perspectives in the context of remyelination by promoting oligodendroglial cell recruitment to the lesion and controlling in an AMPK-dependent manner the maturation of OLs until axon engagement.

Thus, GSA-10 might constitute the first member of a new generation of selective and potent remyelinating agents.

## Data Availability Statement

The raw data supporting the conclusions of this article will be made available by the authors, without undue reservation.

## Ethics Statement

The animal experiments were performed in accordance with the Council Directive 2010/63EU of the European Parliament and approved by the French Ethic Committees (CEEA59 and CEEA26). Written informed consent was obtained from the owners for the participation of their animals in this study.

## Author Contributions

AR-W: conceptualization and supervision: *in vitro* studies. ET and MRa: conceptualization and supervision of animal study. AR-W, ET, and MRa: funding acquisition and project administration. AR-W, ET, MRa, AD, MRs, LT, HF, AK, SB, CS, and FB: design of the experiments and writing of the original draft. AD, AR-W, ET, MRa, MRs, LT, HF, and AK: perform the experiments and analyzed the data. AR and FB: design and produce the microfiber supports. AD, MRs, and AK: visualization. All authors have read and agreed to the published version of the manuscript.

## Conflict of Interest

The authors declare that the research was conducted in the absence of any commercial or financial relationships that could be construed as a potential conflict of interest.

## Publisher’s Note

All claims expressed in this article are solely those of the authors and do not necessarily represent those of their affiliated organizations, or those of the publisher, the editors and the reviewers. Any product that may be evaluated in this article, or claim that may be made by its manufacturer, is not guaranteed or endorsed by the publisher.

## Footnotes

1
http://www.prestwickchemical.com/prestwick-chemical-library.html
