# Supplementary material for: Smoothened/AMP-Activated Protein Kinase Signaling in Oligodendroglial Cell Maturation
Source: Front Cell Neurosci. 2022 Jan 10;15:801704. doi: 10.3389/fncel.2021.801704 (PMC8784884; doi:10.3389/fncel.2021.801704)
Supplement: Supplementary file 1 [file Data_Sheet_1.docx]

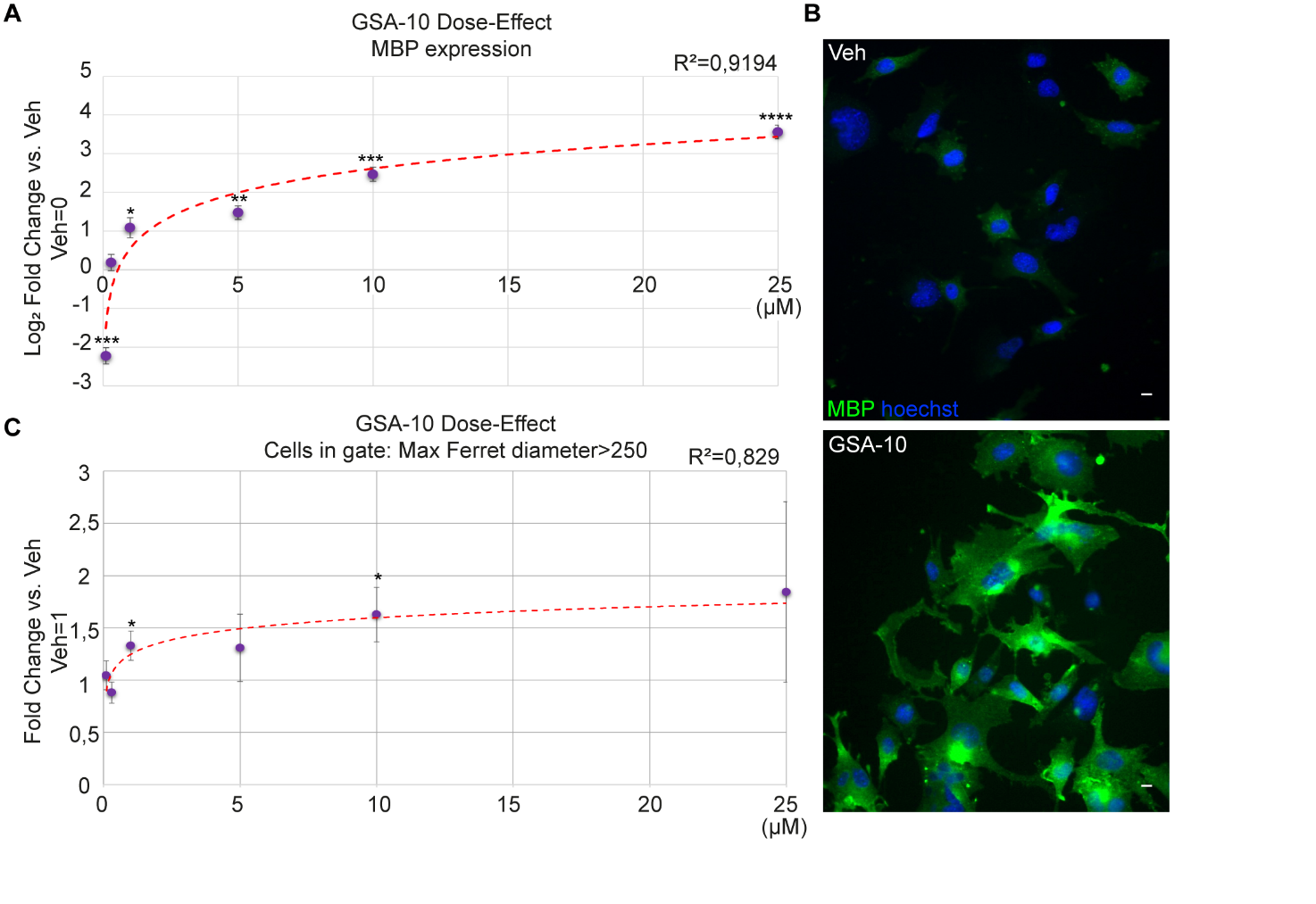


**Supplementary Figure 1.** GSA-10 increases Oli-neuM differentiation to MBP-expressing cells *in vitro*. (**A-C)** Dose-effect curve of GSA-10 in Oli-neuM treated for 48h with a differentiation medium supplemented with GSA-10 at 0.1; 0.3; 1; 5; 10; 25 µM or with the Vehicle. (**A)** Dose effect on MBP gene expression was analysed by quantitative RT-PCR. Data were plotted as log2 fold change versus Vehicle (which was set as 0) ± SEM (n ≥ 3). (**B,C)** Dose effect on membrane enlargement was evaluated by immunostaining with anti-MBP primary and Alexa 488 secondary antibodies (FITC); nuclei are labelled with Hoechst (Blue). Scale bar, 10 μm. Quantitative morphological analysis was performed using ScanR software (Olympus) using Max Feret Diameter and Mean Intensity FITC parameters. (**B**) Typical images of GSA-10 concentration selected, 10 µM. (**C**) Data were plotted in a graph by ScanR software and the mean % population within the gate MaxFeretDiameter > 250 (y)/MeanFITC (MBP) (x) comparatively evaluated to identify the larger membrane expansion. Data are shown as fold induction versus vehicle arbitrarily set at 1± SEM (n ≥ 3). Statistical significance was calculated using GraphPad: two-tailed Student’s t test versus Vehicle. * p < 0.05, ** p < 0.01, *** p < 0.001, **** p < 0.0001. The red lines are the trend lines of the dose curves. The respective R values are indicated in the graphs.


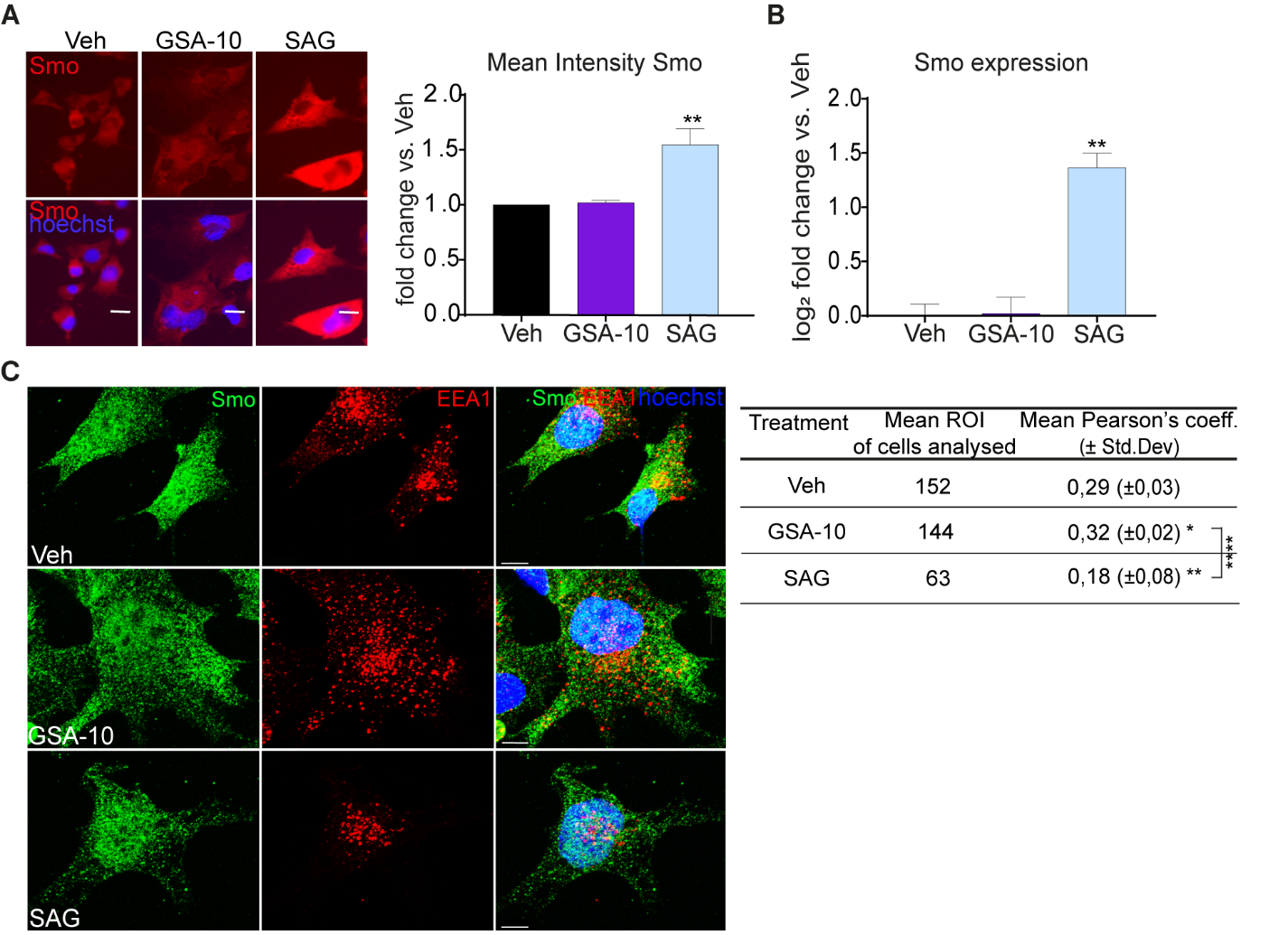


**Supplementary Figure 2.** Analysis of Smo levels and expression in Oli-neuM. (**A)** Quantitative analyses of Smo^+^ IF in Oli-neuM cells treated for 48 h with 5 μM SAG, 10 μM GSA-10, or vehicle. Data were normalized respect to vehicle, arbitrarily set to 1, and plotted as fold change versus vehicle. Values are mean ± SEM (n ≥ 3). Typical images shown were stained with anti-Smo primary and Alexa 546 secondary antibodies (red); Hoechst (Blue) = nuclei. Scale bar, 10 μm. (**B)** Smo expression analysis treated as described in (**A**). Data were normalized and plotted as log2 fold change. Values are mean ± SEM (n ≥ 3) respect to Vehicle. Two-tailed paired Student’s t test was used for statistical significance versus vehicle. One-way ANOVA with Tukey’s multiple comparison test was used to analyse statistical significance among different treatments. ** p < 0.01.


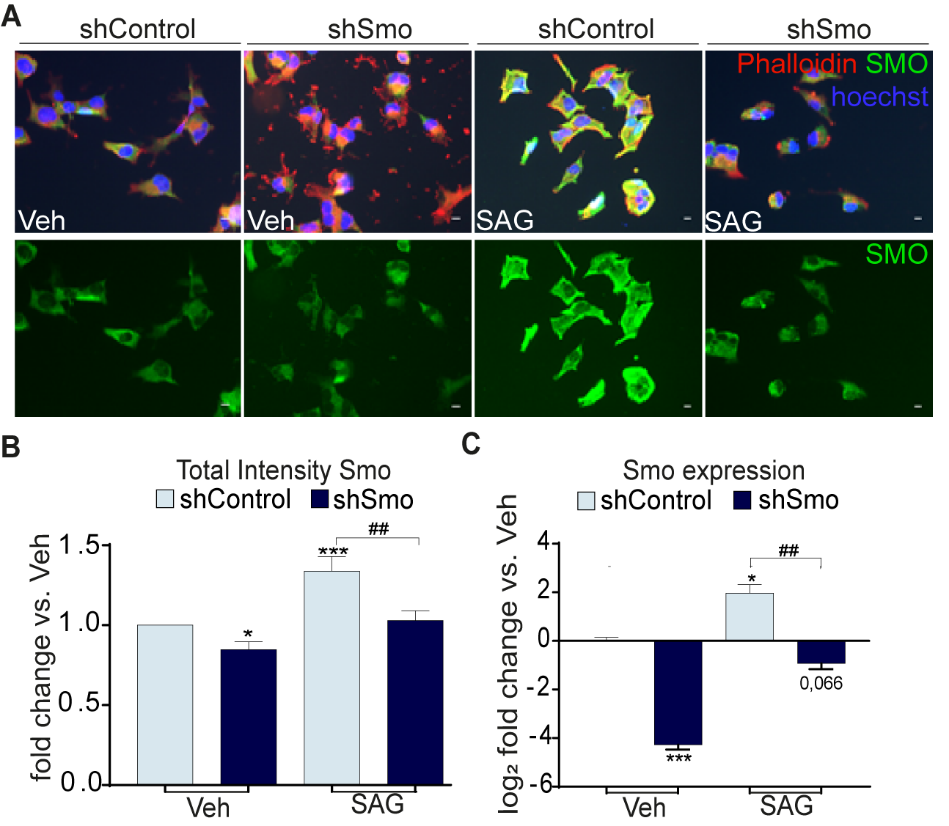


**Supplementary Figure 3.** Smo silencing in Oli-neuM. (**A-B)** Quantitative IF analyses of shSmo cells respect to shControl. Both cell lines were treated for 48h with 5 µM SAG or vehicle. Mean FITC (Smo) intensity was plotted as mean ± SEM (n ≥ 3). Data were normalized respect to vehicle shControl, arbitrarily set to 1. Representative images of shSmo and shControl cells in (**A**) were stained with anti-Smo primary and Alexa 488 secondary antibodies (Green); Phalloidin (Red); Hoechst (Blue) = nuclei. Scale bar, 10 μm. (**C)** The graph shows Smo gene expression level after 48h treatment in shSmo and shControl cells. Data were normalized and plotted as log2 fold change and correspond to mean ± SEM (n ≥ 3) respect to shControl Vehicle, represented as 0. Two-tailed paired Student’s t test was used for statistical significance versus shControl vehicle (* labelled) and to compare shControl vs shSmo (# labelled) in each treatment. One-way ANOVA with Tukey’s multiple comparison test was used to analyze statistical significance between the different treatments. * p < 0.05, *** p < 0.001 ; ## p < 0.01.
